# Assemblages of carabid beetles (Coleoptera, Carabidae) in humid forest habitats of different stages of succession in the Puszcza Knyszyńska Forest (northeastern Poland)

**DOI:** 10.3897/zookeys.100.1539

**Published:** 2011-05-20

**Authors:** Adam Kwiatkowski

**Affiliations:** Laboratory of Evaluation and Assessment of Natural Resources, Warsaw University of Life Sciences – SGGW, Warsaw, Poland

**Keywords:** Carabidae, Puszcza Knyszyńska, process of succession, wet and humid, forest habitats, Mean Individual Biomass

## Abstract

During a period of three years (2006–2008) the carabid fauna in wet and humid forest habitats of different stages of succession was studied at the Puszcza Knyszynska (north-east part of Poland). The aim of this study was to determine how the assemblages of the carabid fauna change in relation to the ongoing process of succession. Using pitfall traps, 24 plots were sampled. The plots were located in stands of different age, from two year old plantations to more than 100 year old forests. Additionally, the stands were ordered in three moisture classes (wet, humid and very humid) and two classes of soil richness. As indicators for change in the carabid fauna in relation to age of the stands Mean Individual Biomass (MIB), species diversity and share of forest species were used. By applying multivariate statistics the relation of the different habitat characteristics to changes in the carabid fauna was examined. During the study 8903 individuals belonging to 57 species were collected. *Pterostichus niger* represented 28% of the total catches and therefore the most common species. Another common species, *Pterostichus melanarius*, contributed to 13% of the total catch. This species was caught at every plot, even in the old forests. In contrast to the results obtained by Szyszko (1990) for fresh and dry pine stands, in this study the relation of MIB with the age of forest was not significant. Although the number of species was rather constant, the number of individuals belonging to the group of forest species significantly increased with the ageing of the forest. The multivariate analysis showed a relationship with ageing of the stands and soil richness rather than with moisture and size of the forest. According to the present paper, clear cuttings in wet and humid habitats do not cause a strong degradation of the carabid fauna.

## Introduction

Carabid beetles represent one of the largest groups of animals, occurring in almost every type of terrestrial habitat. In Poland over 500 species of these insects are recorded (Burakowski et al. 1973, 1974). The reaction of these animals to environmental changes is fast and pronounced. Habitat changes may be natural due to succession, or anthropogenic due to human management. Therefore, AT each stage of succession, carabid beetles are characterised specific assemblages. Consequently, this group of animals can be used as an indicator of environmental changes (Rainio and Niemelä 2003, Pearce and Venier 2006).

Szyszko (1990) used the Mean Individual Biomass (MIB) of carabid beetles as an indicator of successional changes in pine forests growing in dry and fresh habitats. However, data concerning wet and humid forest habitats are lacking. Therefore, a study on carabid fauna in this type of habitat was carried out over a period of three years (2006 – 2008) in the Puszcza Knyszyńska forest. This forest is located in the north-eastern part of Poland and it is characterized by many small forest rivers. In the valleys of the forest rivers and streams, wet and humid forests on organic and very rich soil have developed. In the past, forests growing in this type of habitat were exploited using clear cuts. In the last few years single clear cuts were restricted to an area of 2–3 ha. Therefore, the study sites, where the research was carried out, usually are no larger than 3 ha. Nowadays, the forest service tries to avoid using clear cuts, because this type of forest management is thought to have negative impact on the environment (Dyrektor Generalny Lasów Państwowych 1999). On the other hand, a clear cut can be, to a certain degree, compared to a natural catastrophe (e.g. wind break, fire), which arrests the process of succession. After a clear cut, secondary succession starts from a comparably young stage. With increasing age of the forest stand, advanced stages of succession develop at various speeds (Szyszko 1990). Studies on carabid beetles (see Rainio and Niemelä 2003) show that these animals react quickly after disturbance of their environment. After a clear cut, species characteristic of forests disappear, whereas species typical for open habitats rapidly colonise the area. However, with ongoing succession, typical forest species reappear and their share consequently increases (Szyszko 1990, Koivula and Niemelä 2002, Paquin 2006).

The aim of the study was to analyse how the assemblages of carabid beetles occurring in wet and humid forest habitats change with an ongoing process of succession (age of the forest stands). These changes were expected to be analogous to those observed in other forest types as pine forests (Szyszko 1990) and boreal mixed-wood forests (Jacobs et al. 2008). Therefore, the main hypothesis is that with preceding succession the characteristics of the carabid fauna change from those typical for open land to those typical for forest habitats. Accordingly, MIB values as well as the share of forest species, autumn breeders and large zoophages, which are supposed to predominate in mature forests (Skłodowski 2006), should significantly increase, but the number of species should decrease.

However, besides age of the forest stands, other factors like environmental conditions or habitat fragmentation (e.g. Desender 2005) may have an influence on the development of the carabid assemblages. Therefore, a second aim was to analyze the impacts of age of size of the forest stand, as well as its moisture and soil richness on the carabid assemblages.

## Methods

**Figure 1.**
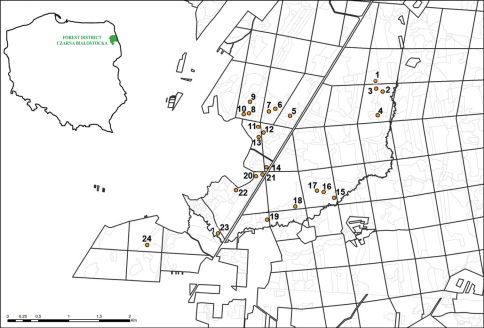
Location of the sampling plots in the Puszcza Knyszyńska forest. For specifications, see Table 1.

The study was carried out in 24 sampling plots (Fig. 1), in the Puszcza Knyszyńska forest, located in the forest district Czarna Białostocka (north-eastern Poland). The sampling plots were located in independently treated units of the forest of different age and size, at least 50 m apart. The age of the units ranges from a 2 year old plantation after clear cut to over 100 year old forests and size ranges from about 1 ha to almost 11 ha. Additionally, the units were classified with respect to moisture conditions and soil richness (Table 1) accordingly to the periodical inventories of forest habitats and forest resources done for the forest district Czarna Białostocka (Biuro Urządzania Lasu i Geodezji Leśnej Białystok 2006). Based on these data, moisture of the sampling plots was assessed on a scale of three grades: 1 – humid plots, 2 – wet plots, 3 – marshy plots. Soil richness was assigned to two levels: 1 – rich soil (mixed deciduous forest), 2 – very rich soil (deciduous forest).

**Table 1.** Characterisation of the studied forest units with respect to size, age, moisture and soil richness. See text for explanations.

*Number of sampling plot*	*Size (ha)*	*Age (years)*	*Moisture*	*Soil richness*
1	2.09	68	1	1
2	7.16	73	2	1
3	1.87	91	1	1
4	1.59	4	1	2
5	1.11	13	1	2
6	2.82	7	3	2
7	1.91	127	3	2
8	2.39	3	3	2
9	4.35	46	1	2
10	2.02	93	3	2
11	3.35	28	3	2
12	1.64	29	1	2
13	7.46	81	1	2
14	1.05	22	2	1
15	2.09	8	2	1
16	2.35	3	1	1
17	10.72	83	2	1
18	2.9	26	3	2
19	1.86	46	1	1
20	2.2	3	2	1
21	1.06	103	2	1
22	2.07	93	2	1
23	1.72	78	3	2
24	6.51	101	1	1

Carabid beetles were collected using pitfall traps (Barber 1931), with modifications according to Szyszko (1981). Jar glasses were used as traps. A funnel with an upper diameter of about 10 cm and a lower diameter of about 1.6 cm was placed above them flush with the soil surface. A roof was installed a few centimeters above the funnel and ethylene glycol was used as trapping fluid.

At each sampling plot three pitfall traps were situated 5 m apart. Collection of carabids was carried out from 2006-2008, from mid-May to the end of September and traps were emptied three times during this period. Carabid beetles were identified using the keys of Hurka (1996) and Müller-Motzfeld (2004). The nomenclature follows Hurka (1996).

For each sampling plot the following parameters of the carabid fauna were calculated: number of collected species, share of individuals of typical forest species, share of individuals of big zoophages, share of individuals of autumn breeding species (Lindroth 1945; Burakowski et al. 1973, 1974; Hurka 1996; Müller-Motzfeld 2004) and MIB value. MIB values were calculated by dividing the biomass of all sampled carabids by the number of specimens caught. Biomass values were fixed for the recorded species using values from Szyszko (1990) or, with respect to species not included in his paper, using the formula by Szyszko (1983) that describes the relationship between the body length of a single carabid individual (x) and its biomass (y):

ln *y* = -8.92804283 + 2.55549621 ´ ln *x* (eq. 1)

Correlations between these parameters and the age of the forest sites were tested using the Spearman rank correlation coefficient (Sachs 1984).

To analyse the impact of the factors size, age, soil richness and moisture of the sampling plots, a multivariate analysis (RDA) was carried out using the CANOCO for Windows software package, v. 4.5 (ter Braak 1987; ter Braak and Šmilauer 2002). Detrended Canonical Correspondence Analysis (DCCA) was carried out in advance to select the appropriate response model (ter Braak and Prentice 1988). Redundancy Analysis (RDA) was performed using inter-species correlations and dividing by standard deviation. Data were not transformed. Centering was done only by species. No forward selection was carried out. A triplot was created with species fit range adjusted from 25 to 100 % (passed by 15 species).

## Results

During the three years of investigation 8903 individuals, belonging to 57 species of carabid beetles, were collected (Table 2). Seven of these protected by law in Poland (Ministerstwo Środowiska 2004). Being the most common species, *Pterostichus niger* contributed 27.8% to the catches. Another very common species,e catches. Another very common species, with a percentage share of 13.7%, was *Pterostichus melanarius*. Irrespective of the age of the stands, this species was recorded from each plot,.

**Table 2.** List of collected species and their ecological characteristics concerning habitat preference, breeding type and feeding type. Species are sorted by total abundance and dominance (%) (nomenclature according to Hurka 1996).

*Species*	*Habitat preference*	*Breeding type*	*Feeding type*	*Total abundance*	*Dominance (%)*
*Pterostichus niger*	F	A	B	2472	27.78
*Pterostichus melanarius*	O	A	B	1219	13.70
*Pterostichus oblongopunctatus*	F	S	S	599	6.73
*Oxypselaphus obscurus*	F	S	S	589	6.62
*Patrobus atrorufus*	F	A	S	486	5.46
*Carabus granulatus* *	E	S	B	414	4.65
*Carabus hortensis* *	F	A	B	389	4.37
*Epaphius secalis*	E	A	S	308	3.46
*Pterostichus aethiops*	F	S	S	285	3.20
*Carabus coriaceus* *	F	A	B	269	3.02
*Cychrus caraboides*	F	A	B	246	2.76
*Europhilus fuliginosus*	F	S	S	198	2.22
*Pterostichus strenuus*	F	S	S	164	1.84
*Carabus glabratus* *	F	A	B	159	1.79
*Agonum afrum*	F	S	S	142	1.60
*Platynus assimilis*	F	S	S	92	1.03
*Calathus micropterus*	F	A	S	86	0.97
*Carabus nemoralis* *	F	S	B	80	0.90
*Carabus arvensis* *	F	S	B	67	0.75
*Pterostichus anthracinus*	O	S	S	60	0.67
*Poecilus versicolor*	O	S	S	54	0.61
*Pterostichus nigrita*/*Pterostichus rhaeticus*	E	S	S	51	0.57
*Badister lacertosus*	F	S	H	44	0.49
*Bembidion mannerheimi*	O	S	S	44	0.49
*Notiophilus palustris*	F	S	S	37	0.42
*Harpalus latus*	E	A	H	35	0.39
*Pterostichus vernalis*	E	S	S	31	0.35
*Stomis pumicatus*	E	A	S	31	0.35
*Clivina fossor*	O	S	S	29	0.33
*Loricera pilicornis*	E	S	S	29	0.33
*Agonum sexpunctatum*	E	S	S	24	0.27
*Harpalus quadripunctatus*	F	S	H	24	0.27
*Carabus cancellatus* *	O	S	B	22	0.25
*Pterostichus minor*	E	S	S	17	0.19
*Badister sodalis*	O	S	H	16	0.18
*Bembidion lampros*	O	S	S	14	0.16
*Leistus terminatus*	F	A	S	12	0.13
*Amara familiaris*	E	S	H	9	0.10
*Pseudoophonus rufipes*	O	A	H	9	0.10
*Synuchus vivalis*	E	A	S	8	0.09
*Amara lunicollis*	E	S	H	5	0.06
*Leistus piceus*	F	A	S	5	0.06
*Amara communis*	O	S	H	3	0.03
*Amara plebeja*	E	S	H	3	0.03
*Pterostichus diligens*	F	S	S	3	0.03
*Dyschirius globosus*	E	S	S	3	0.03
*Amara curta*	O	S	H	2	0.02
*Harpalus luteicornis*	E	S	H	2	0.02
*Nebria brevicollis*	F	A	S	2	0.02
*Notiophilus biguttatus*	F	S	S	2	0.02
*Amara similata*	O	S	H	2	0.02
*Agonum viduum*	E	S	S	2	0.02
*Amara brunnea*	F	A	H	1	0.01
*Harpalus anxius*	O	S	H	1	0.01
*Anisodactylus binotatus*	E	S	H	1	0.01
*Amara nitida*	O	S	H	1	0.01
*Platynus krynickii*	F	S	S	1	0.01

* - species protected by law in PolandHabitat:

**F** – typical forest species.

**E** – eurytopic species.

**O** – open habitat speciesBreed:

**S** – spring breeder.

**A** – autumn breederFood:

**B** – large zoophage.

**S** – small zoophage.

**H** – hemizoophage

**Figure 2.**
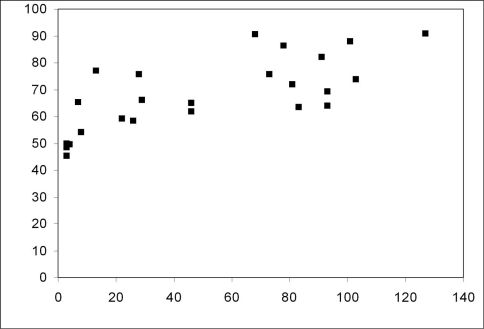
Relationship between the contribution of forest species and the age of sampled forest stands (Spearman rank correlation coefficient *rs*=0.696; *p*=0.001)

**Figure 3.**
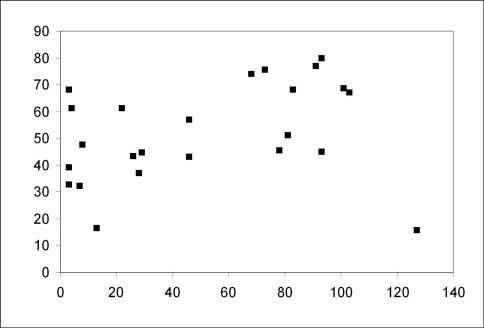
Relationship between the contribution of large zoophages and age of the sampled forest stands (Spearman rank correlation coefficient *rs*=0.485; *p*<0.05)

With increasing age of the forest stands the share of forest species increased significantly from below 50% in the young stands to almost 90 % in the older stands (Fig. 2; Spearman rank correlation coefficient *rs*=0.696, *p*<0.001). The share of large zoophages also increased significantly (Fig. 3; Spearman rank correlation coefficient *rs*=0.485, *p*<0.05), whereas the share of autumn breeders did not show a significant increase with the age of the stands (Spearman rank correlation coefficient *rs*=0,371, n.s.). Species numbers remained rather constant with increasing age of the forest stands (Spearman rank correlation coefficient *rs*=0.18, n.s.). No significant correlation between MIB values and the age of the stands was observed (Spearman rank correlation coefficient *rs*=0.111, n.s.).

**Figure 4.**
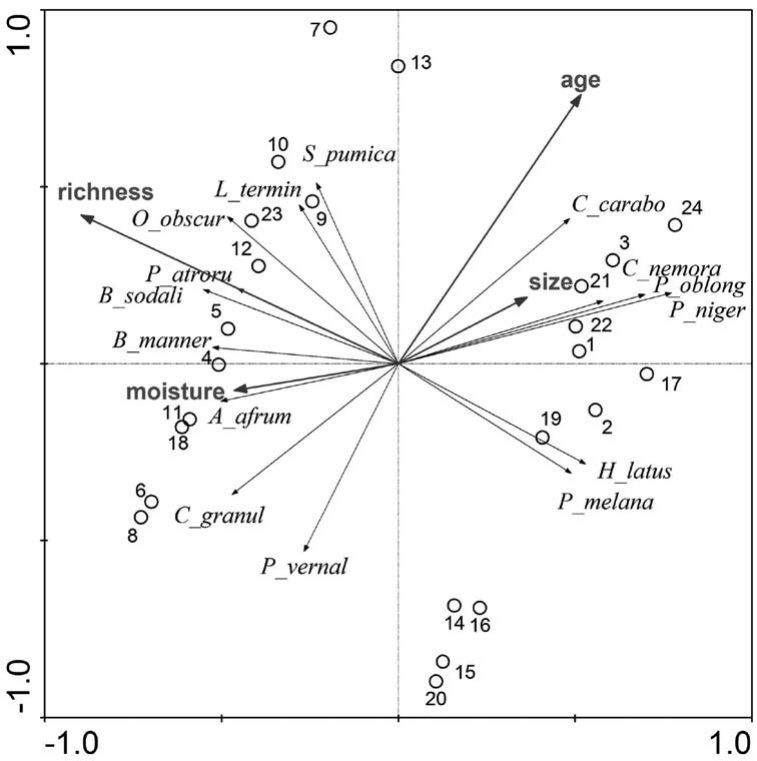
Multivariate analysis (RDA) carried out with the dataset (number of the sampling plots as in Table 1). See text for explanations.

The first two axes of the RDA explain 36.2% of the variance in the species data and 91.5% of the variance in the fitted species data. The diagram (Fig. 4) indicates a stronger impact of age of the stands and soil richness, whereas the impact of moisture and size of the forest stands is less strong. The latter has the lowest impact on the structure of the carabid assemblages among the four studies factors. Age of the forest stand and soil richness are not correlated. Accordingly, the sampling plots are located along the first and second ordination axes concerning their soil richness and age. Sampling plots with very rich soil are located to the left of the diagram, whereas sampling plots of less rich soil are located to the right of the diagram. Younger sampling plots are located in the lower part and the older sampling plots are located on the upper part of the diagram.

## Discussion and conclusion

The main hypothesis of the study was only partly confirmed. The contribution of forest species and large zoophages to the carabid fauna increased significantly with age of the forest stands, but that of autumn breeders, number of species and MIB values did not. Age of the forest stands and soil richness were more important regarding the structure of carabid assemblages, whereas moisture and size of the forest stands appeared to be of less importance.

This increase of the contribution of forest species and large zoophages was expected, since it was observed in other studies as well, for example pine forests and boreal mixed-wood forests (Szyszko 1990, Schwerk 2008, Jacobs et al. 2008). After disturbance of a forest environment, which arrests succession, the number forest specialists rapidly decreases (Szyszko 1990, Koivula and Niemela 2002, Paquin 2006). The observed increase in share of typical forest species and large zoophages shows that with ongoing succession the carabid fauna is changing from species typical for open areas to a typical forest fauna.

However, the contribution of autumn breeders and species numbers did not change significantly, the latter contradicts the results found for the succession in pine forests (Szyszko 1990). Also the increase of MIB values with the age of stands was not significant. (Szyszko (1990, 1991) presented MIB as simple and good indicator that describes the stages of succession in pine forests growing on dry and fresh soil. In humid and wet forests this indicator seems to be less useful, the process of succession is slower or the impact of clear cuttings is less sever in humid and wet habitats.

The results of the study indicate that the degree of degradation and the process of regeneration of the carabid fauna in wet and humid forest habitats on organic soil is somewhat different compared to habitats of dry pine stands. This might be due to the fact that the forests of the present study grow on very rich, organic soil. After a clear cut in this type of forests, the method of soil preparation before planting young trees is different from forests on dry and mineral soil. The soil of the forests studied in the presented paper was never ploughed. However, ploughing destroys the litter layer of the habitat, which is necessary for the occurrence of many species characteristic of forests (Skłodowski 2006, Pihlaja et al. 2006). In the present study almost all MIB values for young sampling plots exceed 100 mg, which indicates that the degradation of the habitat by clear cutting and soil preparation was comparatively low. MIB values for young stages of succession in fresh pine forests often amount to about 40 mg (Schwerk 2008). The assumption of a low degradation of the studied forest sites is also supported by the fact that soil richness and age of the sampling plots are not correlated in the RDA analysis.

In the Puszcza Knyszyńska forest, clear cuts in wet habitats are usually no larger than 3 ha. Even if the size of the forest seems to have a comparatively low impact on the structure of the carabid assemblages in the present study, the size and shape of clear cut areas may be important factors. In order to support species diversity, it seems to be necessary to avoid creating large-sized open areas by clear cutting in large forest complexes. In this context the dispersal power of the respective species is important. For example, according to Spence et al. (1996) beetles characteristic of old forests may be not be able to recolonize patches of uncut forests if the distance between suitable patches becomes too large. On the other hand, clear cuts create suitable conditions for species with preferences for open areas (Skłodowski 2002, Koivula and Niemelä 2002). As many of these species are able to fly, these areas can be colonized rapidly even when located inside large forest complexes, for which also forest-roads can be used (Koivula and Niemelä 2002). In the present study twelve species of open areas were recorded only in young plantations of an age of 2–5 years.

Despite some areas of clear cuts, the Puszcza Knyszyńska forest as a whole can be regarded as an ancient woodland. However, these clear cuts may contribute to fragmentation of the ancient forest, thus being a threat for forest species diversity. Assmann (1999) and Desender (2005) showed that for forest species with a low dispersal power the size of ancient forests is most important. Therefore, excessive fragmentation of ancient forests may lead to the extinction of local populations of species specialized on forests (Desender 2005). In the present study *Carabus glabratus*, which according to Assmann (1999) is a characteristic species of ancient woodlands, was recorded from 15 of the 24 sampling plots. This suggests that the applied practice of small sized, narrow clear cuts seems not to affect species diversity in the Puszcza Knyszyńska forests much.
